# Composition, isolation, identification and function of adipose tissue-derived exosomes

**DOI:** 10.1080/21623945.2021.1983242

**Published:** 2021-11-16

**Authors:** Rui Zhao, Tiantian Zhao, Zhaozhao He, Rui Cai, Weijun Pang

**Affiliations:** Laboratory of Animal Fat Deposition & Muscle Development, College of Animal Science and Technology, Northwest A&f University, Yangling, China

**Keywords:** Adipose tissue-derived exosomes, composition, isolation, identification, cancer, cardiovascular diseases

## Abstract

Exosomes are nano-sized extracellular vesicles (30–160 nm diameter) with lipid bilayer membrane secrete by various cells that mediate the communication between cells and tissue, which contain a variety of non-coding RNAs, mRNAs, proteins, lipids and other functional substances. Adipose tissue is important energy storage and endocrine organ in the organism. Recent studies have revealed that adipose tissue-derived exosomes (AT-Exosomes) play a critical role in many physiologically and pathologically functions. Physiologically, AT-Exosomes could regulate the metabolic homoeostasis of various organs or cells including liver and skeletal muscle. Pathologically, they could be used in the treatment of disease and or that they may be involved in the progression of the disease. In this review, we describe the basic principles and methods of exosomes isolation and identification, as well as further summary the specific methods. Moreover, we categorize the relevant studies of AT-Exosomes and summarize the different components and biological functions of mammalian exosomes. Most importantly, we elaborate AT-Exosomes crosstalk within adipose tissue and their functions on other tissues or organs from the physiological and pathological perspective. Based on the above analysis, we discuss what remains to be discovered problems in AT-Exosomes studies and prospect their directions needed to be further explored in the future.

## 1. Introduction

**Figure 1.**
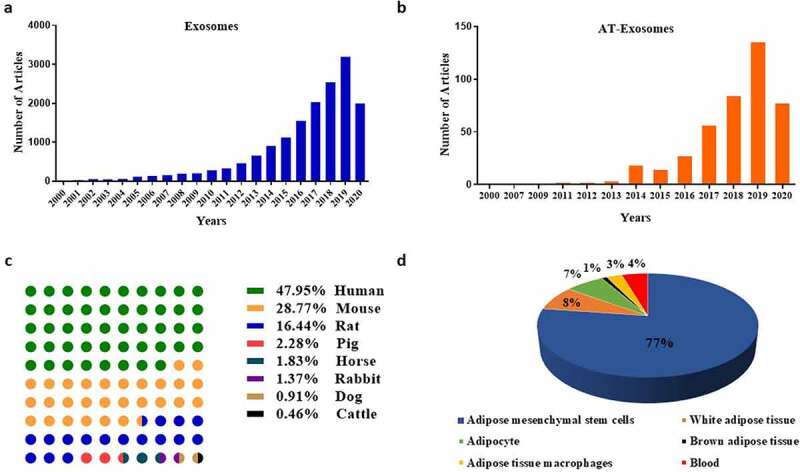
Classification and statistics of articles related to exosomes and AT-Exosomes. (a). Number of exosomes literatures along with year. (b). Number of AT-Exosomes articles along with year. (c). The literatures of AT-Exosomes in different species including human, mouse, rat, pig, horse, rabbit, dog and cattle. (d). Source of AT-Exosomes. AT-Exosomes, adipose tissue-derived exosomes

Adipose tissue (AT) is a main energy storage organ, which is a loose connective tissue with stromal vascular fraction (SVF), adipose-derived stem cells (ADSCs), adipose tissue macrophages (ATMs), T cells, preadipocytes and mature adipocytes. Literatures have shown that AT, as an endocrine organ, secretes a variety of adipokines that affect the body’s homoeostasis, including leptin, adiponectin, resistin and adipsin [1–3]. In terms of biogenesis, exosomes are a kind of extracellular vesicles (EVs). EVs are heterogeneous membrane vesicles that have been proven to be secreted by all types of cells [4]. They contain various types of particles with a wide range of physical properties and biological origins, being mainly divided into two categories based on different sizes [5]. One is 50–1000 nm vesicles formed by the sprouting of the plasma membrane, mainly including ectosomes (ie, shedding vesicles, microparticles and microvesicles) and apoptotic bodies, the other is exosomes with a size of 40–160 nm from the endosomal membrane. In physiological and pathological conditions, different types of cells secrete exosomes into the extracellular environment and body fluids [6], including blood [7], urine [8], amniotic fluid [9], spermatozoa [10]. Exosomes have a wide range of functions related to biological processes, including transfer of functional proteins and nucleic acids [11], immune response [12], elimination of unwanted substances, nutrition [13], surface receptor-mediated cell signalling [14] and cancer metastasis [15–17]. We use the keyword ‘exosome’ to search for relevant literature in the NCBI PubMed Database (https://pubmed.ncbi.nlm.nih.gov/), observing that exosomes-related literatures have risen sharply in recent years (Figure 1(a)). Studies on the biological origin, transport and function of exosomes will broaden the understanding of unknown intercellular communication and tissue homoeostasis in a mammal. Due to the lipid bilayer membrane structure, exosomes protect their coated substances and target specific cells or organs. Furthermore, exosomes have been used as disease biomarkers [18] and therapeutic agents [19] for disease diagnosis and drug carrier studies.

In recent years, AT has been proven to play an important physiological function by releasing AT-derived exosomes (AT-exosomes) to other specific tissues and organs. Its hypertrophy changes the miRNA profile of exosomes in plasma and affects glucose absorption and lipid metabolism in mice [20]. The exosomes derived from adipocytes (Adipocytes-Exosomes) affected the mTOR signal of the hypothalamus to regulate the appetite and body weight of mice [21]. Moreover, exosomes secreted by human adipose tissue mesenchymal stem cells (ADSCs-Exosomes) had a positive effect on the treatment of Alzheimer’s disease [22]. They were confirmed to affect the growth and migration of liver cancer, ovarian cancer, breast cancer [23–25]. We use the keywords ‘adipose’ and ‘exosome’ to search and classify relevant kinds of literature in the NCBI PubMed database. The studies of AT-Exosomes show an upward trend year by year similar to exosomes (Figure 1(b)). Additionally, by the analysis of the functions of different species and cell sources, we find that ADSCs-Exosomes are the most concerned by researchers (Figure 1(c.d)), because ADSCs are pluripotent cells that are easy to obtain and are cultured in large quantities with clinical therapeutic potential. In the above studies, the extraction and identification methods of AT-Exosomes are the research basis. Furthermore, studies about the internal components of AT-Exosomes help us make better use of this biological endogenous delivery to play a variety of regulatory functions. In this review, we summarize the components of AT-Exosomes, the methods of isolation and identification, as well as their physiological and pathological functions.

## 2. AT-Exosome composition

Given the universality and particularity of exosomes cargoes in adipose tissue, it was divided into two parts. The composition of universal substances is mainly caused by the biogenesis of exosomes, including some membrane proteins and key proteins of vesicle formation, which are contained in all of the exosomes. Specific substances are mainly molecules related to the metabolites secreted by adipose tissue and lipid storage, without in other tissue. In this section, we summarize the universal and specific components of AT-Exosomes.

### 2.1. Universality composition

The universality of exosomal composition is largely determined by its biogenesis. The biological process mainly includes the reverse budding of cytoplasmic membrane to form multivesicular endosome, the fusion of mature endosome and cell membrane, and the secretion of exosomes to the extracellular environment [26]. According to the origin and biogenesis of exosomes, all exosomes contain proteins involved in membrane transport and fusion (GTPases, annexins, flotillin) [27–30], tetraspanin proteins (CD9, CD63, CD81, CD82) [30–33], Heat shock proteins (Hsc70, Hsp90) [33,34], vesicle-forming proteins (Alix, TSG101) [30,33], lipid-associated proteins and phospholipases [35]. This has also become an important marker to identify exosomes [21,36,37]. In addition to proteins, exosomes contain lipids such as cholesterol, ceramides, sphingolipids, and long-chain glycerophospholipid, as well as a variety of RNAs including mRNA, miRNA, other non-coding RNAs (snRNA, snoRNA, scaRNA, piRNA, lncRNA and circRNA), tRNA and rRNA. Interestingly, most of the RNAs in exosomes are 20–200 nt in length.

### 2.2. Specificity composition

The specificity of the internal components of AT-Exosomes is reflected in their different sources of species and cells, determining their different biological functions (Table 1). The gluteal fat releases exosomal Hotair (HOX transcript antisense RNA) that promoted the proliferation of intestinal stem cells and progenitor cells [38]. In addition, AT-Exosomes are rich in lipids, lipid-related mRNAs and proteins [39–43]. Cargoes of AT-Exosomes have functions similar to AT and affect lipid synthesis and homoeostasis of target organs and cells. ADSCs-Exosomes contained miR-125a which acted on endothelial cells and promoted angiogenesis [44]. ADSCs-Exosomes were shown to secrete exosomes containing miR-4792, miR-320b, and miR-320a to inhibit the viability of ovarian cancer cells [11]. Moreover, ADSCs-Exosomes were detected to be rich in lncRNA metastasis associated lung adenocarcinoma-transcript 1 (MALAT1), lncGm37494 and other lncRNAs to perform a variety of important physiological functions [45–51], mainly including enhancing the regeneration of neurons in the injured area, repairing spinal cord injury, stimulating wound healing, angiogenesis, and improving hypoxia-induced cardiac injury. Furthermore, the exosomal microRNA-34a secreted by adipocytes inhibited the polarization of M2 macrophages and promoted fat inflammation caused by obesity [52]. Besides, adipocytes-Exosomes circRNA circ-DB to target ubiquitin-specific protease 7 (USP7) to promote the growth of hepatocellular carcinoma [53]. Our laboratory has devoted itself to the function of the ingredients in AT-Exosomes. The previous study indicated that resistin-containing exosomes secreted from adipocytes caused fatty degeneration of the liver in mice [54], suggesting that AT-Exosomes are new potential targets for the treatment of obesity and related hepatorenal syndrome.

**Table 1.** Cargo in AT-exosomes in different species

Species Cargo	Human	Mouse	Rat	Pig	Cattle
microRNA	miR-125a [44], miR-4792, miR-320b, miR-320a [11], miR-145 [158]	miR-27a [55], miR-155 [56,145], miR-92a [57], microRNA-34a [52]	miR-214 [58], miR-191 [59], miR-450a-5p [60], miR-126, miR-130a, miR-132, miR-let7c [61]	miR-148a, miR-532-5p, miR-378, let-7 f [39]	miR-142-5p [40]
lncRNA	lncRNA MALAT1 [45–50] anti-NOS2a, DLG2A5, GAS5, HOTAIRM1, lincRNAp21, lincRNA-VLDLR, NEAT1 [50]	LexGm37494 [51], Hotair [38]			
circRNA		circ-DB [53], circ-0001359 [62], circ-0000250 [63]			
tRNA	tRNA CTC [79]				
mRNA				ANXA4, CLTC, CCT2 [64], MDFIC, HGF, CEBPA, ARRB1 [39]	CPT1A, HSL, PLIN, ATGL, FABP4 [40]
protein	IL6 [65], NEP [66], PTRF [67], Alpha-1-Antitrypsin [68]	FASN, G6PD, ACC [41], aP2 [42], eNAMPT [69], resistin [54]	caveolin 1, AQP7, adenylate kinase 2, catalase, liver carboxylesterase [70]		

**Figure 2.**
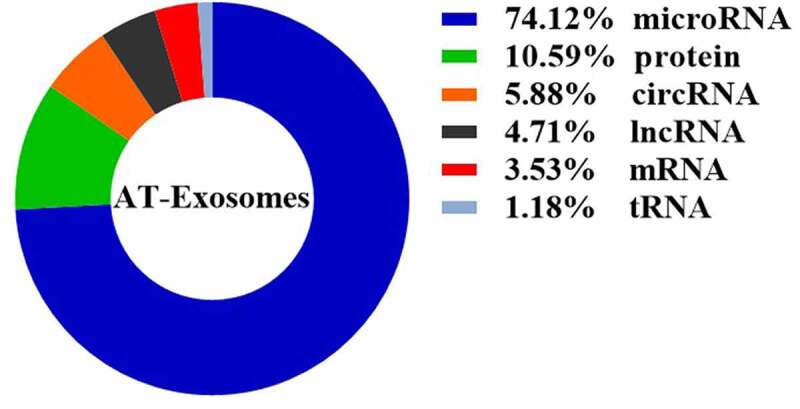
The proportion of components in AT-Exosomes

We conduct a statistical analysis of the AT-Exosomes in the retrieval articles mentioned above (Figure 2), finding that the studies on microRNA components accounted for 74.12%. This is mainly due to the short length of microRNA and the complete microRNA sequence in exosomes, which makes it easy to perform biological functions. Furthermore, the current study methods including RNA-sequencing analysis, proteomics and lipidomics related to exosomal composition are limited. From a long-term perspective, the components of AT-Exosomes need to be further supplemented. It will also help us better understand the principles of exosomes and the communication between cells and tissue, this endogenous biological medium to perform physiological and pathological functions.

## 3. AT-Exosome isolation

In recent years, studies on the function of AT-exosomes have become a hot spot. The basic method is successfully isolates and detect them without disturbing natural form. In this part, we briefly summarize the principles and methods of several commonly used AT-Exosomes separation, and further analyse their respective advantages and disadvantages (Table 2).

**Table 2.** The features of AT-Exosomes isolation methods

Characteristics based on	Method	Instrument requirement	Separation time	Shape & structure	Concentration	Purity	References
Size	Differential ultracentrifugation	high	long	destroy	normal	normal	[71–73]
	Gradient ultracentrifugation	high	long	protect	low	high	[84,85]
	Ultrafiltration	normal	short	destroy	normal	normal	[43,62,74]
	Size-exclusion chromatography	high	short	protect	high	normal	[75,95]
Water soluble	Polymer precipitation	low	short	destroy	high	low	[46,76,147]
Marker protein	Immunoaffinity capture	low	short	destroy	high	low	[108]

### 3.1. Ultracentrifugation

Ultracentrifugation is called the ‘gold standard’ for exosome extraction according to the different sedimentation coefficients of exosomes and other substances [77,78]. It is mainly divided into differential ultracentrifugation and density gradient ultracentrifugation.

#### 3.1.1. Differential ultracentrifugation

The differential ultracentrifugation is based on the size of exosomes. It is suitable for the extraction of exosomes in various body fluids [79–82]. The differential centrifugation method has the advantages of simple operation and low cost. However, the exosome samples prepared by this method have high levels of protein aggregation and lipoprotein contamination [83], and the purity is low. This method requires high sample volume and equipment requirements. In addition, ultra-high-speed centrifugation may cause exosome fragmentation, which is not conducive to subsequent quantitative and functional studies.

#### 3.1.2. Gradient ultracentrifugation

The gradient ultracentrifugation is based on the size and density of exosomes. Density gradient centrifugation is mainly a combination of differential centrifugation and density centrifugation [84,85]. The principle is that exosomes will be suspended in liquids of similar density or composition after centrifugation. Exosomes have been reported to have a density between 1.13–1.19 g/ml [86]. The most commonly used solvents are mainly sucrose and iodixanol. The method of sample primary centrifugation is consistent with gradient centrifugation. The difference is that, 30% sucrose or iodixanol should be added to the bottom of the centrifuge tube before ultracentrifugation, and ultrahigh-speed centrifugation of 100,000–120,000 × g should be used for at least 75 min. In the next step, the suspension is resuspended by adding pre-chilled PBS, centrifuged at 100,000–120,000 × g and washed again, and the resulting precipitate (exosomes) is resuspended in a solvent according to different subsequent needs.

The density of the exosomes extracted by density gradient centrifugation is high in purity, which avoids protein contamination to a certain extent and protects the morphology of exosomes [87]. However, this method isolates exosomes with EVs similar to exosomes size, reducing the production of exosomes and separation efficiency.

#### 3.1.3. Ultrafiltration

The separation of exosomes by ultrafiltration is similar to the traditional filtration principle, mainly using ultrafine nano-membranes with different MWCO (molecular weight cut-off) to separate extracellular vesicles of different sizes [88–90]. The main process of extracting exosomes by ultrafiltration is relatively simple. Various body fluids and cell culture supernatants are sequentially passed through filters to remove small particles such as cell debris, free proteins, and large vesicles such as apoptotic bodies, and collect exosomes smaller than 200 nm.

Compared with the ultracentrifugation method, the ultrafiltration method greatly shortens the experimental time, and has lower requirements for experimental equipment. However, this method easily causes vesicle congestion on the nanofiltration membrane to damage the membrane. Pressurization during filtration also destroys the natural form and structure of the exosomes, causing exosomes rupture and reducing the recovery rate [88,91]. In addition, exosomes separated based on size alone are likely to be mixed with nanoparticles of comparable size, resulting in exosome contamination and affecting exosomes purity [92].

### 3.2 Size-exclusion chromatography

The highlighted advantage of this separation method is that it does not destroy the structure and integrity of exosomes [93]. Under this separation method, the exosomes are observed to be normal in structure and the size of vesicles is about 80–200 nm. After treatment of the cells, there is no significant reduction in cell viability [94]. Furthermore, the characteristic of no interaction with **a**nother recently reported method for separating exosomes based on size is size-exclusion chromatography (SEC), also known as gel chromatography, molecular exclusion chromatography, etc., which is a type of liquid chromatography [95–97]. The separation mechanism of exclusion chromatography is three-dimensional exclusion, and there is no interaction between the sample components and the stationary phase. The packing material of the chromatographic column is a gel, which is an inert surface and contains many pores or three-dimensional network materials of different sizes. When the sample to be separated passes through the chromatographic column, the components of different sizes can penetrate into the gel pores at different depths. The large component molecules cannot enter the small pores or even be completely repelled and are quickly eluted, then the separated exosomes are obtained.

The stationary phase ensures a high separation efficiency [92,98]. Generally, we recommend that the separation method of size exclusion chromatography is more suitable for the separation, identification and subsequent functional study of exosomes. However, this method has higher requirements on instruments, showing wide size distribution of exosomes after isolating. Meanwhile, there are contaminants similar in size to exosomes, including protein aggregates and lipoproteins. Therefore, some researchers have used it in conjunction with ultrafiltration to remove possible contaminants [98–100].

### 3.3. Polymer precipitation

The polymer precipitation method mainly uses highly hydrophilic polymers to interact with water molecules around the exosomes to form a hydrophobic microenvironment, thereby allowing the exosomes to settle down [101,102]. The currently used hydrophilic polymers are mainly polydiethanol [103]. First, centrifuge at 3000 × g for 15 min to remove cell debris and apoptotic bodies, and aspirate the supernatant. Aspirate the supernatant and filter with a 0.22 μm filter. The suspension and the hydrophilic polymer solution were incubated overnight at 4°C. Then, centrifuge at a speed of 1500 × g for about 15 min, and discard the supernatant. The resulting precipitate (exosomes) will be resuspended in a solvent according to different subsequent needs.

The advantages of the polymer precipitation method are mainly simple operation, high yield and no need for complicated equipment. Therefore, it is often used in the production of commercial kits [47,104,105]. However, the polymer also precipitates various water-soluble substances in the exudate, including nucleic acids, lipoproteins, proteins, and even viruses. Thereby, it may lead to a high probability of exosome contamination by this method [106], causing great difficulties for the subsequent morphological observation and functional exploration of exosomes.

### 3.4. Immunoaffinity capture

The immunoaffinity capture method mainly uses specific proteins on the surface of exosomes for separation. As mentioned above, all exosomes contain some specific proteins and cell membrane components, so they can be used for the capture of exosomes based on immunoaffinity [107]. This includes proteins involved in membrane transport and fusion (GTPases, annexins, flotillin), four types of transmembrane proteins (CD9, CD63, CD81, CD82), heat shock proteins (Hsc70, Hsp90), vesicle-forming proteins (Alix, TSG101) and lipid-associated proteins and phospholipase. Existing institutions have used this feature to develop kits for isolating exosomes based on different markers.

Similar to the polymer precipitation method, the immunoaffinity capture method is also simple to operate and does not require expensive experimental machinery [108]. Simultaneous separation and extraction take a short time. Non-physiological pH-worthy eluents are used during the operation of this method, so it inevitably destroys the physiological structure of exosomes and affects the subsequent functional studies. Additionally, immunocapture of specific proteins is limited to exosomes with known antigens and may be affected by the heterogeneity of exosomes [109].

We have analysed and summarized the methods of AT-Exosomes separation in a number of studies which is special for adipose tissue, the extraction process are as follows: (1) Centrifuge at a low speed (300 × g) for about 10 min to remove impurities and live cells in the sample (this part should be noted that when separating the body fluids of high concentration exosomes such as plasma, use pre-chilled PBS first dilution). (2) Collect the supernatant, centrifuge at 2000 × g for 10–20 min, and centrifuge at 10,000 × g for 30–40 min. This process is to remove dead cells and cell debris. (3) Aspirate the supernatant, filter at 0.22 μm and enter into ultracentrifugation. (4) The filtered liquid is centrifuged for at least 70 min by ultra-high speed of 100,000–120,000 × g, discard the supernatant, and resuspend the pellet in PBS. (5) 100,000–120,000 × g centrifugation to wash the precipitate, and then resuspend the obtained precipitate (exosomes) using solvents according to different needs.

## 4. AT-Exosome identification

According to the structure, size and formation process of exosomes, the identification of exosomes currently mainly includes marker protein expression detection mentioned above, electron microscope observation and nanoparticle tracking analysis (NTA). Of course, these methods were also used to identify AT-Exosomes.

### 4.1. Marker protein detection

Based on the formation process and principle of exosomes mentioned above, exosomes have a series of specifically expressed proteins. In these years of research, a large number of experimenters have used this feature to carry out preliminary auxiliary identification of the isolated and extracted exosomes [110–114]. Among them, the specific proteins commonly used for detection mainly include CD63, CD9, TSG101 and Alix.

### 4.2. Electron microscope observation

Electron microscope observation is mainly divided into transmission electron microscope and cryo-electron microscope. Under the transmission electron microscope (TEM), the exosomes show a saucer-like structure, and its size is roughly judged to be between 40–160 nm [115]. Exosome samples are first fixed with 2% paraformaldehyde. Suspended droplets are taken on the carbon membrane copper mesh and dried to be negatively stained with dye [115–118]. Do not need to undergo complicated TEM sample preparation operations such as fixation, dehydration, embedding, and ultrathin sectioning [119], but instead directly stain the sample homogenate suspension. The negative staining technique is not only simple and fast but also the amount of dyeing solution is very small and the resolution is high.

However, some researchers point out that the saucer-like structure under the transmission electron microscope of exosomes is most likely caused by collapse after drying [120,121] Meanwhile, the fixed staining in TEM sample preparation and vacuum observation during imaging may also affect the size and morphology of exosomes [122]. In contrast, the exosomes observed by cryo-electron microscopy are round, which seems to be more consistent with the shape of the organism [123,124]. The preparation of the low-temperature electron microscope sample is that the exosome suspension is directly adsorbed on the porous carbon grid, sucked dry at 95%–99% humidity and quickly immersed in liquid ethane. This process does not require fixation and staining and is observed at −180°C, which maximizes the size and shape of the isolated exosomes. Therefore, more and more researchers choose to use this method to identify and semi-quantitate exosomes [125–127]. To ensure the staining effect and exosomal morphology, it is necessary to carefully select dyes and grasp the staining time.

### 4.3 Nanoparticle tracking analysis

NTA is a technology developed based on the principle of light scattering and Brownian motion of particles in suspension [128,129]. It detects the concentration and size distribution of vesicles with a diameter of 10 nm–1 μm and has recently been used for quantitative detection of exosomes [130–132]. The minimum size of vesicles using traditional flow detectors is about 500 nm, and a few improved detectors can only detect particles of 200 nm [133], which is not in line with the small size of ‘40–160 nm’ unique to exosomes. In the NTA experiment, a laser beam is used to pass through the sample chamber, and the exosomes suspend in the beam path scattered the light, making it easy to see them through a 20× magnification microscope equipped with a camera. The camera runs at 30 frames per second (fps) and captures video files of particles [134]. From the collected video records, the displacement of each particle is tracked and plotted as a function of time, and its hydrodynamic diameter is calculated using the Stokes-Einstein equation. After calibration with microspheres of known concentration, the absolute size distribution of the vesicles in the suspension can be obtained [135].

The detection cycle of NTA technology is very short, and it can measure more than 1000 particles in only 60 s [136]. In addition, NTA has 405 nm, 488 nm, 532 nm and 635 nm lasers with four different wavelengths to choose from, with corresponding filters, so that it can analyse fluorescent samples. There are specific markers on the surface of exosomes, including CD63, HSP70 and TSG-101 [137,138]. Under complex background conditions, using fluorescent antibodies to label exosomes, researchers can use the fluorescence measurement function of NTA to measure exosomes, and the results are more reliable than flow cytometry [139,140].

However, NTA testing also has certain limitations. When there are a large number of large-sized vesicles in the sample, the overlapping may affect the identification and tracking of small vesicles by the instrument. Consistent with this, the same problem occurs when the exosomes concentration in the resuspension solution is too large [130]. Therefore, this technique requires the concentration of exosome suspension to be controlled between 10^8^–10^9^ /mL [141,142]. Thereby it needs a good grasp of the density of the exosomal suspension, and sample pretreatments such as dilution or concentration if necessary.

Above all, we introduce three methods to obtain AT-exosome better, but none of them is perfect. Most of the studies use the combination of differential ultracentrifugation, marker protein detection, electron microscope observation, and NTA to isolate and identify exosomes. So far there are no methods to identify specific AT-exosomes, but we can make full use of the multi-omics to exploit the specific marker protein, thereby identifying the AT-exosomes in the future. Furthermore, with the development of the technology, it is possible to identify the specific exosomes. SP-IRIS, with higher sensitivity and accuracy, can characterize the particle size difference exosomes from different sources. Daniel et al use the SP-IRIS to explore the size difference of the exosomes from serum, finding that the exosomes from CD9 were ~10 nm larger than those from other sources [143]. Further SP-IRIS research still needed to be performed to uncover the methods which can identify AT-exosomes specifically.

## 5. Function of AT-Exosomes

**Figure 3.**
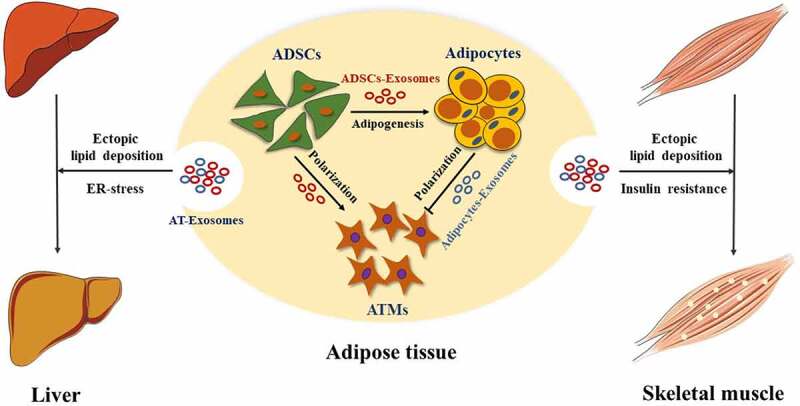
AT-Exosomes modulate ATMs polarization and adipogenesis in adipose tissue, liver and skeletal muscle. ADSCs, adipose tissue mesenchymal stem cells. ATMs, adipose tissue macrophages. ER-stress, endoplasmic reticulum stress

**Figure 4.**
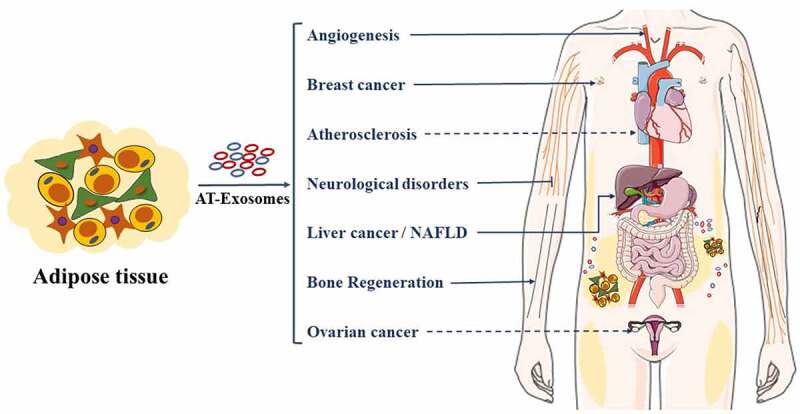
AT-Exosomes control human cancers and other diseases. AT-Exosomes may arrive target organs mainly through blood circulation. NAFLD, Non-alcoholic fatty liver

As the largest energy storage and secretory organ, adipose tissue has attracted more and more attention to its secretion and function of exosomes. By classifying statistics of research papers retrieved from the database, we summarize the important roles of AT-Exosomes in both physiological and pathological aspects (Figures 3 and 4).

### 5.1. AT-Exosomes physiological function

#### 5.1.1. AT-Exosomes function within adipose tissue

The heterogeneity of cells in adipose tissue determines that there will be crosstalk between cell exosomes in adipose tissue. The hypertrophy of adipose tissue causes the infiltration and activation of ATMs, which further affects body weight and produces insulin resistance. Deng et al first found AT-exosomes and showed that AT-exosomes of obese mice activated ATMs, leading to increased production of proinflammatory cytokines IL-6 and TNF-α in 2009. This process enhanced the migration of ATMs to adipose tissue and liver and promotes the development of insulin resistance [84]. Adipocytes-Exosomal miRNA-34a secreted by adipocytes inhibited M2 macrophage polarization and promoted obesity-induced fat inflammation [52]. Melatonin acted on ATMs by promoting the secretion of exosomes by adipocytes, reducing fat inflammation [116]. Further, Adipocytes-Exosomes contained lipid droplets and were absorbed by ATMs [43]. Additionally, in the white adipose tissue of diet-induced obese mice, ADSCs-Exosomes promoted the polarization of M2 macrophages and reduced the inflammatory response in mice [144]. Meanwhile, ATMs also released miRNA-rich exosomes to act on fat cells, liver cells and skeletal muscle cells, affecting the body’s metabolic homoeostasis [145]. In summary, ADSCs, adipocytes and ATMs in adipose tissue use exosomes as a biological medium to interact with each other to jointly maintain metabolic homoeostasis (Figure 3). However, AT-Exosome research is still needed to further reveal the underlying mechanisms and specific signalling molecules and pathways in adipose tissue.

#### 5.1.2. AT-Exosome function in liver

The liver is an organ with complex functions in the organism, playing a vital physiological role in metabolism, detoxification, digestion, lipid synthesis and storage. Adipose and liver tissue interact with hormones and other biologically active factors to jointly maintain the body’s homoeostasis [146]. A study has shown that exosomal miRNA derived from brown adipose tissue (BAT) affected liver gene expression. Under cold stress conditions, miR-132-3p in exosomes secreted by BAT promoted liver fat production [147]. Melatonin reduced the transport volume of Adipocytes-Exosomal resistin from adipocytes to liver cells, thereby further alleviating liver fatty degeneration caused by endoplasmic reticulum stress [54]. Interestingly, ADSCs promoted the release of liver exosomes [148], and the miR-130a-3p derived from liver exosomes reduced glucose tolerance by targeting PH domain leucine-rich repeat protein phosphatase 2 (*PHLPP2*) in adipocytes [36]. Taken together, these findings indicate that there is indispensable exosome-mediated crosstalk between adipose tissue and the liver, revealing the potential of exosome-based therapies to control liver fat production and further reduce liver steatosis (Figure 3).

#### 5.1.3. AT-Exosome function in skeletal muscle

As the largest organ of mammals, skeletal muscle is responsible for basic functions including exercise, breathing and metabolism. Studies have shown that metabolic disorders of adipose tissue affected the metabolism of fatty acids and the release of adipokines, further causing ectopic lipid deposition in skeletal muscle [149]. Adipocytes-Exosomes were regarded as a new type of adipokine, which affects the lipid metabolism in skeletal muscle [150]. 3T3-L1 Adipocytes-Exosomal miR-27a was shown to inhibit insulin signalling in C2C12 cells through PPARγ. Interestingly, ATMs-exosomal miR-155 also promoted skeletal muscle insulin resistance through PPARγ [145]. PPARγ knockout in adipocytes attenuated the thickening of the myocardium caused by miR-200a in exosomes [151]. It further suggests that adipose tissue and skeletal muscle are in close communication by their exosomes (Figure 3).

### 5.2. AT-Exosomes pathological function

#### 5.2.1. AT-Exosomes and tumours

AT-Exosomes play an important role in the growth and migration of liver cancer, ovarian cancer, breast cancer and other cancers (Figure 4). ADSCs-Exosomes promoted the growth and migration of hepatocellular carcinoma [152]. Mechanistically, miR-23a/b and circ-DB were transported into liver cancer cells via ADSCs-Exosomes, thereby promoting the growth and migration of liver cancer cells [23,53]. Moreover, some researchers have proposed that ADSCs-Exosomes mediated exogenous transport of miR-199a-3p and miR-122 to increase the chemical sensitivity of hepatocellular carcinoma [153,154]. ADSCs-Exosomal miR-21 transferred to ovarian cancer cells, inhibiting the apoptosis of ovarian cancer cells by binding to the target apoptotic protease activating factor-1 (*APAF1*) and giving them chemoresistance [155]. However, ADSCs-Exosomal microRNAs inhibited the proliferation of ovarian cancer cells and promoted their apoptosis [16]. ADSCs-Exosomes also promoted the migration of breast cancer cells [156]. Global gene expression profile analysis showed that breast cancer cells treated with ADSCs-Exosomes upregulated cancer-related signalling genes and activated Wnt signalling pathways. In addition, ADSCs-Exosomes targeted inducible costimulatory molecule to promote anti-tumour immunity of lung adenocarcinoma [157], increased collagen beta (1-O) galactosyltransferase 2 (*COLGALT2*) expression to promote osteosarcoma proliferation and diffusion [17], inhibited prostate cancer cells proliferation, and induced prostate cancer cell apoptosis through miR-145 [158]. As mentioned above, AT-Exosomes have different effects on the proliferation, apoptosis and differentiation of different cancer cells. Furthermore, for the same type of cancer cell, the effects of different components in AT-Exosomes on the recipient cells are also inconsistent. In summary, ADSCs-Exosomes have been shown to effectively promote the proliferation or migrate of certain cell types and significantly reduce the proportion of apoptotic cells. Additional work is needed to enhance the use of AT-Exosomes in tumour therapy and confirm the optimal concentration for human use, as it will increase the feasibility and safety of AT-Exosome therapy in clinical applications.

#### 5.2.2. AT-Exosomes and obesity-related diseases

AT-Exosomes have been shown to affect diabetes, non-alcoholic fatty liver disease (NAFLD) and cardiovascular diseases induced by insulin resistance (Figure 4). AT-Exosomes induced insulin resistance by activating ATMs polarization [84]. In this process, ATM-exosomal miR-210 regulated glucose uptake and mitochondrial activity, thereby promoting the onset of obesity and diabetes in mice [159]. Evidence showed that BAT-Exo significantly mitigated the metabolic syndrome in HFD mice by being involved in catalytic processes to promoted oxygen consumption in recipient cells [160]. Obesity changed the miRNA profile of plasma exosomes in mice, including the up-regulation of miR-122, miR-192, miR-27a-3p and miR-27b-3p, causing glucose intolerance and insulin resistance [161]. Additionally, cystatin C levels in AT-Exosomes were positively related, and monocyte marker CD14 levels were negatively related to metabolic complications of obesity, being used as a potential marker for cardiovascular-related diseases [162]. As a common obesity-related metabolic disease, the effect of ss-Exosomes on atherosclerosis was controversial. But more details about the molecular mechanism must be elucidated. ADSCs-Exosomes played a role in promoting atherosclerosis by regulating the formation and polarization of macrophage foam cells [163], whereas ADSCs-Exosomes protected endothelial cells from atherosclerosis [164]. Exosomes from visceral adipose tissue integrated into liver cells and induced dysregulation of TGF-β pathway members *in vitro* and offered an intriguing possibility for the pathogenesis of NAFLD [165]. Although AT-Exosomes can be used as diagnostic markers and therapeutic targets for obesity-related metabolic diseases. Currently, there are more and more studies on ADSCs-Exos related to obesity, and more studies are needed to investigate the understanding of exosome functions in obesity.

#### 5.2.3. AT-Exosomes and angiogenesis

Angiogenesis is a key biological process that affects development, skeletal muscle hypertrophy, menstruation, pregnancy and wound healing (Figure 4). It mainly relies on the extensive signal transduction network formed by endothelial cells, parietal cells, vascular smooth muscle cells, pericytes and immune cells. ADSCs-Exosomes have been reported in numerous articles related to angiogenesis. Studies showed that ADSCs-exosomes stimulated the proliferation and migration of microvascular endothelial cells to promote angiogenesis by platelet-derived growth factor [166]. Mechanically, ADSCs-Exosomes was proved to be rich in small RNAs including miR-125a, miR-199-3p, miR-181b-5p and miR-423-5p, affecting the proliferation and migration of endothelial cells by binding their target genes [44,167–169]. In addition, hypoxia-induced ADSCs-Exosomes improved angiogenesis through activating the PKA signalling pathway and upregulating the expression of vascular endothelial growth factor (*VEGF*) [170]. In conclusion, although there are few effective clinical treatments for angiogenesis at present, cell-free therapies such as wound healing may be a valuable tool in promoting recovery after injury.

#### 5.2.4. AT-Exosomes and bone regeneration

AT-Exosomes play an important role in the repair and regeneration of bone tissue and affect the proliferation, apoptosis, differentiation, ageing of osteoblasts, inducing bone damage caused by osteoarthritis and age-related bone loss (Figure 4). ADSCs-Exosomes and 3T3-L1 Adipocytes-Exosomes promoted the proliferation and osteogenic differentiation of human primary osteoblasts and 3T3-L1 precursor adipocytes [171,172]. And the exosomes derived from human adipose stem cells (hADSCs-Exo) The results indicate that hADSCs-Exo could be absorbed by hADSCs and induce osteogenic differentiation at 15 μg/ml [173]. Above all, they ensure optimal concentration which could promote the proliferation and migration of hADSCs. After ADSCs were pretreated by TNF-α, their therapeutic effect was much better. Moreover, ADSCs-Exosomes enriched in miR-375 improved the osteogenic differentiation of human bone marrow mesenchymal stem cells [174]. Meanwhile, ADSCs-exosomes inhibited the inflammation and oxidative stress of osteoblasts [175] and upregulated the expression of miR-145 and miR-221 [176] to promote cartilage production, improve the anti-ageing effect of osteoblasts and repress osteoarthritis. ADSCs-Exosomes alleviated osteocyte apoptosis and osteocyte-mediated osteoclastogenesis induced by hypoxia/serum deprivation [177]. Low-dose laser irradiation promoted ADSCs-Exosomes therapeutic effect [178]. ADSCs-Exosomes contained specific substances that induced osteogenic differentiation of cancer stem cells, being used to reprogram cancer stem cells into non-tumorigenic cells [179]. Recent studies indicated that hydrogel loaded with ADSCs-Exosomes promoted the bone regeneration in rat skull defect models, providing a basis for clinical applications [174]. Currently, AT-Exosomes-related studies on bone regeneration are rare and more investigations are still needed to expand our understanding of exosome functions in bone tissue.

#### 5.2.5. AT-Exosomes and neurological disorders

AT-Exosomes have the potential to treat neurological diseases (Figure 4). ADSCs-Exosomes improved the survival and proliferation of neurons after injury [46]. Mechanistically, ADSCs-Exosomal MALAT1 mediates protein kinase C δ II (PKCδII) splicing, thereby improving the survival rate of neurons. ADSCs-Exosomes significantly enhanced neurite outgrowth *in vitro*, and ultraviolet radiation reduced the effect of ADSCs-Exosomes on neurite outgrowth [180]. After being internalized by Schwann cells (SCs), ADSCs-Exosomes significantly promoted SCs proliferation, migration, apoptosis, myelination and secretion of neurotrophic factors [181,182]. Additionally, ADSCs-Exosomes inhibited the activation of microglia by the NF-kB and MAPK pathways, reducing the cytotoxicity of activated microglia and preventing neuroinflammation [183]. Some researchers proposed that ADSCs-Exosomes may be used as an effective treatment tool for tissue engineering nerves, increasing the growth of neurites and enhancing regeneration of the sciatic nerve *in vivo* [184]. However, more details about the molecular mechanism must be elucidated, and clinical trials of the regeneration of the nerve must be conducted. Moreover, miR-133b exogenously modified ADSCs-Exosomes promoted the recovery of nerve function in animals with spinal cord injury by affecting signal pathways related to axon regeneration [104].

#### 5.2.6. AT-Exosomes and other disease

AT-Exosomes have the potential to treat other diseases. Evidence shows that exosomes derived from MuSCs control important immunomodulatory effects to protect acute colitis induced by DSS [185]. Although the potential for treating acute colitis is significant, additional work is needed to confirm the optimal concentration. Furthermore, ADSCs-Exo also regulates the recovery after injury. Liu et al isolated the exosomes from mesenchymal stromal cell and added to the TSCs, the result showed that ADSCs-Exo could be absorbed by TSCs and activated the SMAD2/3 and SMAD1/5/9 pathways to promote the proliferation, migration [186]. ADSCs-exosomes also could promote hair follicle regeneration in vivo [187]. Nevertheless, even more details about the underlying molecular mechanism should be explored. Evidence suggests that ADSCs-Exo regulates the regeneration of the myelin sheath by reducing autophagy of injured SCs via miRNA-26b which could downregulate the expression of Kpna2 [188]. Above all, the function of adipose-derived exosomes is very powerful. At present, the research on the function of exosomes is further deepened, and more studies will focus on the study of exosomes in the future.

## 6. Future outlooks in AT-Exosomes

AT-Exosomes are media produced internally by organisms, which have good protection for their contents and target specific organs and cells to exert biological functions. This review introduces the research progress and universal and specific cargoes of AT-Exosomes. To explore the biological function of AT-Exosomes, we summarize the principles and procedures of main methods for the isolation and identification of AT-Exosomes. Importantly, we focus on the effects of AT-exosomes on organism tissues or organs in physiology and pathology.

As mentioned above, the methods of separating AT-Exosomes have their advantages and disadvantages. To date, ultracentrifugation is still regarded as the ‘gold standard’. Moreover, through the analysis of the separation time, cost and purity of various methods, we recommend size exclusion chromatography in addition to ultracentrifugation and commercial kits based on water-soluble extraction. It has the advantages of low cost and short separation time but also has problems of low purity and serious pollution. Therefore, new separation methods also should be developed to maximize the purity of AT-Exosomes and maintain their shape and activity. The physiological functions of AT-Exosomes are currently limited to tracking *in vivo* and phenotypic investigations, and the mechanism of crosstalk between AT-Exosomes and other tissues or organs is unclear. Therefore, exosome research still needs to be performed to further uncover the underlying mechanisms and specific signalling molecules and pathways. Pathologically, ADSCs-Exosomes have been currently used as therapeutic materials. However, target-specific organs, biological safety and inter-species rejection of ADSCs-Exosomes need to be further explored. Meanwhile, to date, the number of organ diseases that can be effectively treated by AT-Exosomes are limited. More studies are needed to explore this promising area. Future studies on the function of AT-Exosomes will not only help us better understand the crosstalk between mammalian different tissues and organs, but also are expected to fully use their biological functions for related cancer diagnosis and diseases treatment.

## Date availability statement

Included as appendix 1and 2.
